# Factors influencing implementation of simulation in nursing and midwifery training in Malawi

**DOI:** 10.4102/hsag.v29i0.2422

**Published:** 2024-05-15

**Authors:** Gertrude Mwalabu, Annie Msosa, Ingrid Tjoflåt, Christina F. Risa, Patrick Mapulanga, Bodil Bø, Kristin H. Urstad, Masauko Msiska

**Affiliations:** 1 Department of Adult Health Nursing, School of Nursing, Kamuzu University of Health Sciences, Lilongwe, Malawi; 2 Department of Quality and Health Technology, Faculty of Health Sciences, University of Stavanger, Stavanger, Norway; 3 Department of Caring and Ethic, Faculty of Health Sciences, University of Stavanger, Stavanger, Norway; 4 Library Department, Kamuzu University of Health Sciences, Lilongwe, Malawi; 5 Department of Public Health, Faculty of Health Sciences, University of Stavanger, Stavanger, Norway; 6 Institute of Nursing, Faculty of Health Studies, VID Specialized University, Oslo, Norway; 7 Department of Biomedical Sciences, School of Life Sciences and Allied Health Professions, Kamuzu University of Health Sciences, Lilongwe, Malawi

**Keywords:** simulation, nursing, midwifery, education, educators, clinical teaching, Malawi

## Abstract

**Background:**

The study explored factors influencing implementation of simulation-based education (SBE) in nursing and midwifery education in Malawi.

**Aim:**

This study aimed to identify factors influencing nursing and midwifery educators in selected training institutions and clinical sites.

**Setting:**

The study covered one district and four central hospitals, five professional training institutions, Ministry of Health and Nurses and Midwives Council of Malawi officials.

**Methods:**

Using mixed-methods approach, quantitative data were gathered from 293 participants, including 149 final-year nursing and midwifery students, and 144 clinical instructors. Qualitative data were obtained from 24 faculty members, 11 clinical instructors and two key informants. Researchers conducted 37 in-depth interviews, 10 focus group discussions and eight desk reviews. Descriptive statistics were used to analyse the quantitative data, while content analysis was used for qualitative findings.

**Results:**

Five themes emerged from qualitative data: absence of simulation in regulatory body syllabi, insufficient formal training, demand for knowledgeable clinical instructors, inadequate human and material resources, and resistance to change. Survey results indicated that 83% of the participants had theoretical SBE knowledge but lacked practical skills, with only 13% considering SBE as a current teaching method. Educators emphasised lack of infrastructure, skills laboratories, teaching hospitals, equipment, and a deficit in formal training as critical barriers to SBE implementation.

**Conclusion:**

The study concluded that skilled educators, appropriate infrastructure and resources could facilitate SBE implementation in Malawi.

**Contribution:**

Recommendations included regulatory body support, formal training for educators, utilisation of low-fidelity simulators, and establishment of SBE centres and corners in health facilities.

## Introduction

The use of simulation as a pedagogic method is increasingly being adopted in delivering health-related education, including nursing and midwifery curricula globally (Girzelska et al. 2019; Janse van Vuuren, Goon & Seekoe 2018; Li et al. 2022). Simulation-based education (SBE) is a practical group-based approach that improves nursing and midwifery student knowledge and abilities to provide safe patient care and is a valuable strategy for acquiring nursing and midwifery skills (Guinea et al. 2019). It involves the use of high- and low-fidelity mannequins, computerised models, virtual reality systems, standardised patients and other simulators to recreate real-life scenarios in a safe and controlled environment (Evans & Taubert 2019). Simulation-based education allows nursing and midwifery students to develop clinical skills, critical thinking abilities and decision-making skills in the same way they could achieve in actual clinical practice (Bø et al. 2022). Kim (2017), among others, states that there is a paucity of literature regarding the implementation of SBE in low-income countries, as it demands advanced facilities, expensive equipment, and experienced faculty and clinical instructors. While some studies indicate that many educators struggle with how to include such an innovative clinical teaching strategy, particularly in the light of faculty shortage and the lack of appropriate equipment (Janse et al. 2018), several other studies attest that SBE may be implemented through less complex technology-based simulation-like scenarios with standardised patients using low-fidelity simulation settings (Tjoflåt, Koyo & Bø 2021; Livingston et al. 2014; Parry & Fey 2019).

Acquiring fundamental clinical skills prepares students to meet competencies required for nurses and midwives. It is crucial for student nurses and midwives to master competency skills, acquire knowledge, exhibit affective attitudes and hone psychomotor abilities for safe, professional practice, clinical guidance and assistance from nursing and midwifery educators (Baghoomian 2014). Nevertheless, educators need to adopt innovative teaching strategies and create teaching and learning environments that can assist students in effectively applying what they have learned in the classroom to a clinical setting to achieve the expected learning competencies and promote optimal patient outcomes. Several studies have authenticated SBE as an innovative clinical teaching strategy that can create clinical experiences outside the clinical or hospital setting (Akalin & Sahin 2020; Svellingen et al. 2021; Woods & Frogge 2017). Using simulation as a clinical teaching and learning strategy in nursing and midwifery education is not new; millions of health-related professionals globally have been trained to be instructors in simulation (Li et al. 2022). However, no relevant literature exists in the Malawian context. Prior global studies on SBE have focused on different disciplines, cadres, levels and experiences of health professionals but lack examination of factors influencing SBE implementation in low-income countries (Higgins et al. n.d.; Johnson, Scott & Franks 2020; La Cerra et al. 2019).

The available literature demonstrates limited experience and documentation of SBE within health education systems in low-income countries, including Malawi (Lewis, Strachan & Smith 2012). The nursing education framework in Malawi is structured to facilitate a symbiotic integration of theoretical knowledge and practical application within the academic curriculum. Students engage in a cohesive blend of theory and practice, underscoring the significance of a comprehensive approach to professional education. Preceding their clinical placements, students undergo foundational learning experiences, particularly in core modules, such as Adult Health Nursing, Child Health Nursing, Community Health Nursing and Mental Health Nursing. These modules collectively serve as bedrock for subsequent clinical exposure.

In addition to the aforementioned clinical modules, students are exposed to a diverse array of subjects aimed at fostering a holistic understanding of healthcare dynamics. Notably, modules in psychology and sociology contribute to the development of a nuanced understanding of the behavioural and social aspects of patient care. Furthermore, the emphasis on language and communication modules ensures that students are equipped with effective communication skills, which are essential components of professional practice. Basic sciences are also integrated into the curriculum to provide students with a foundational understanding of the physiological and anatomical principles underpinning nursing and midwifery practice. From this perspective, more factual data were needed to identify factors that influenced the implementation of SBE in the Malawian context.

## Objective of the study

The study aimed to identify factors influencing the implementation of SBE by nursing and midwifery faculty, clinical instructors, and students in educational and clinical sites in Malawi.

## Research methods and design

### Design

This study used a concurrent mixed-methods design. Concurrent mixed-methods design is a research design that combines both qualitative and quantitative data collection and analysis within the same phase of a study (Creswell & Creswell 2018). This design is employed to provide a more comprehensive and nuanced understanding of a research problem than can be achieved through either qualitative or quantitative methods alone. Qualitative methods provided an in-depth insight into the intricacies of a phenomenon, while quantitative methods allowed for generalisation and the examination of patterns across a larger population (Polit & Beck 2021). The use of multiple methods helped validate findings. The concurrent mixed methods enhanced rigour of the study.

### Study population and sampling strategy

The target population comprised 1345 students and clinical instructors, 643 final-year nursing and midwifery students and 702 clinical instructors. This population was only from the sampled institutions. Nursing and midwifery students were recruited through a formal letter to the office of deans of students in their respective institutions, whereas clinical instructors were recruited through nursing administrators in their institutions. Ten focus group discussions (FGDs) were conducted with separate final-year students from the same population. The final-year students who participated in the questionnaire survey were requested to recuse themselves from the FGDs. Thirty-seven in-depth interviews were conducted with key informants. Table 1 details the study participants, who comprised final-year students, clinical instructors and key informants. The table also shows the data collection methods for each group of participants.

**TABLE 1:** Target population (*N* = 1345).

Region	Study site	Final-year students (*n* = 149)	Clinical instructors (*n* = 144)	Key informants (Deans, Heads of Department and officials from the regulatory body) (*n* = 37)	Final-year students (*n* = minimum of 8 participants per group)
		Questionnaire		In-depth interviews (37)	Focus group discussions (10)
Southern Region	KUHeS – Blantyre Campus	125	-	6	2
	Malawi College of Health Sciences (Bt Campus)	136	-	2	1
	Queen Elizabeth Central Hospital	-	226	1	-
	Zomba Central Hospital	-	101	2	-
	Zomba Mental Hospital	-	22	1	-
Central Region	KUHeS – Lilongwe Campus	184	-	5	2
	Malawi College of Health Sciences (Lilongwe Campus)	41	-	2	1
	Kamuzu Central Hospital	-	225	2	-
	Nkhotakota District Hospital	-	21	2	-
Northern Region	Mzuzu University	86	-	3	1
	St John of God College of Health Sciences	15	-	3	1
	Mzuzu Central Hospital	-	107	3	-
	St Johns College of Health Sciences	56	-	3	2
Lilongwe	NMCM	-	-	2	-

**Total**	**-**	**643**	**702**	**37**	**10**

NMCM, Nurses and Midwives Council of Malawi.

Stratified random sampling was used in the quantitative study. This was performed to ensure representation from different subgroups (strata) within the population. Purposive sampling was used for the qualitative strand using the same population. Qualitative research often uses purposive sampling, in which participants are deliberately selected based on specific criteria that align with the research question (Bryman 2016).

### Inclusion criteria

- Nursing and midwifery students in the final year of their programme and aged 18 years or older
- Clinical instructors and lecturers with more than 6 months of work experience at the selected institutions
- Deans, Heads of Department and nursing administrators occupying the position for more than 6 months
- Curricula for nursing and midwifery programme being implemented in training institutions to check the inclusion of SBE content.

### Exclusion criteria

- Students in years 1–3 of the professional nursing and midwifery programmes
- Students in years 1 and 2 of the nursing and midwifery technician programme
- Students aged less than 18 years
- Clinical instructors with less than 6 months of work experience in institutions.

### Setting

The study was conducted in four public referral hospitals at the tertiary level, one district hospital and five nursing and midwifery training institutions. There are 4 tertiary facilities in Malawi, 29 district hospitals and a total of 17 educational institutions. The selected settings were based on the regional distribution of health facilities targeting referral hospitals and training institutions. Furthermore, the health facilities were chosen because of the high numbers of nursing and midwifery students they receive for clinical placements from various health training institutions. All of these institutions are key to the training of nurses and midwives. One institution offers professional programmes from bachelor to doctoral levels, three offer programmes from certificate to bachelor levels, and one offers a college diploma programme in nursing and midwifery. Four central hospitals offer tertiary healthcare services with a bed capacity of 750 each and one district hospital (secondary level) with 450 bed capacity.

### Data collection

Data collection comprised the administration of a questionnaire for the quantitative work and interviews with a subset of participants for the qualitative work. The research team comprised the principal investigator and two co-principal investigators, all of whom held doctoral degrees in nursing and research. In addition, two doctoral nursing students were part of the research team and three research assistants supported the team. The quantitative strand included clinical instructors and final-year nursing and midwifery students. A questionnaire was used to collect quantitative data. Quantitative data were distributed to nursing and midwifery students and clinical instructors and then collected using an Android application with daily upload to the cloud. The questionnaire was developed by the researchers based on the research objectives and pilot-tested with Year 2 and 3 students from KUHeS and clinical instructors from a nonparticipating institution. Testing the validity and reliability of a questionnaire is essential to ensure that the results obtained from the questionnaire are accurate and trustworthy (Creswell & Creswell 2018). Face and content validity were established for all relevant areas related to the topic by three experts, who reviewed the questionnaire and provided feedback. Inter-rater reliability was established and involved two raters scoring consistent results on the questionnaire after comparing their scores. A Kappa value of 0.8 or greater was considered substantial agreement. A high inter-rater reliability suggests that the scoring process is consistent and reliable, meaning that different raters are likely to arrive at similar conclusions when using the same assessment tool (McHugh 2012).

The questionnaire consisted of a total of 44 items: biodata (4 items), knowledge of SBE use in nursing and midwifery education (11 items), experience with the use of simulation-based education in nursing and midwifery training and practice (7 items), attitudes, competence, motivation for use of simulation and perceived value for learning (7 items), challenges with the use of SBE (4 items) and feasibility and strategies for improving the use of SBE (8 items). Twenty-eight items were closed-ended, and 13 questions were open-ended (13 items). A Likert scale of 1–5 from strongly agree to strongly disagree was used for the close-ended items. Strongly agree was given a score of 5, strongly disagree a score of 1 and neutral a score of 3. The questionnaire was then uploaded to the Android platform. Researchers read the questions and options on the questionnaire and entered the answers from the participants. The questionnaire took 45 min to 60 min to complete.

### Interviews and discussions

Data for the qualitative portion of the study were collected through a checklist of curricula documents through a desk study to check whether SBE was incorporated into the regulatory body curricula documents. Qualitative data were also collected through interview guides for in-depth interviews and FGDs. Individual interviews were conducted by PhD scholars in nursing and midwifery and a project team comprising the principal and co-principal investigators. These interviews were held at the participants’ institutions at a time convenient for the participants, lasted for a maximum of 45 min, and were immediately transcribed verbatim by three research assistants. See Appendix 1 for a list of the questions used to guide the interviews.

Focus group discussions were conducted with nursing and midwifery students and each group comprised 8–10 participants. Discussion groups lasted 60 min and were held at the participants’ institutions at a time convenient for all participants. See Appendix 2 for the prompts used to guide the FGDs. All FGDs were conducted by three research assistants who were holders in the Bachelor of Science in Nursing and Midwifery with more than 5 years of experience in clinical supervision and data collection. The research team kept field notes for both individual interviews and FGDs.

Qualitative and quantitative data complemented each other with quantitative work informing qualitative work. Data from the questionnaire provided a contextual understanding of the factors influencing SBE implementation. The completion of questionnaires preceded the conducting of interviews and FGDs. Qualitative data provided a deeper understanding of the factors involved in SBE implementation in clinical and training institutions in Malawi. The interviews and FGDs were recorded with participants’ permission to ensure the accuracy of the collected data. Information from the desk review was recorded as notes. The desk review focused on examining nursing and midwifery curricula documents at the regulatory body to determine whether the documents submitted to regulators incorporated SBE. The interview and FGD recordings were transcribed verbatim by three trained research assistants who were fluent in English and had experience in SBE. One session was conducted for each FGD, and the number of FGDs was determined by data saturation, during which no new information was generated. All data for the study were collected by the project team from May to July 2022. The project team comprised the principal investigator, project administrator, clinical coordinator and two doctoral nursing students. All members of the research team visited each educational and clinical institution to collect the data.

### Data analysis

Qualitative and quantitative data were analysed separately, followed by a concurrent triangulation process (Creswell & Creswell 2018). Quantitative data were entered into SPSS version 23.0 (IBM 2021). Data analysis was limited to descriptive statistics that involved organising the data into a dataset and checking for missing values, outliers and inconsistencies. The mean and relative frequencies were calculated. Data were presented using tables or charts, highlighting key descriptive statistics.

Qualitative data were analysed using content analysis guided by Graneheim, Lindgren and Lundman (2017), which included data preparation, describing the unit of analysis, selecting specific texts or materials to be analysed, condensing the data, creating condensed versions of the selected text, developing initial codes by identifying key concepts, applying codes to segments that share similar content or meaning, grouping related codes into broader categories based on similarities, identifying overarching themes, comparing and contrasting themes and categories to ensure that they accurately reflect the data, and ensuring the validity and reliability of the analysis through member checking. The relevant data analysed were transcribed interviews, focus group transcriptions and notes from the desk review. The steps involved organising and preparing the data for content analysis, which included transcribing interviews and focus group discussions. Meaning units were identified, condensed, coded and categorised, and then descriptive themes were detailed. The principal investigator and two co-principal investigators coded data from the interviews, and the three research assistants coded data from the FGDs. These steps of the qualitative content analysis process derived meaningful insights from the qualitative data.

### Trustworthiness

The study demonstrated trustworthiness as described by Shenton (2004). Five PhD lecturers from nursing and midwifery lecturers reviewed the entire process of developing themes to ensure rigour and validity. Credibility was ensured by peer debriefing throughout and after the analysis phase. To promote transferability, we thoroughly described the research context and underlying assumptions. We kept a detailed record of the project’s research process to establish dependability. Finally, confirmability was ensured by meticulously reviewing data sources and analytical findings and documenting the decision-making process of the research team.

### Ethical considerations

Ethical approval to conduct the study was obtained from the College of Medicine Research and Ethics Committee (P.07/21/3362). Permission to access participants was obtained from Central and District Health Offices, educational institutions and clinical training sites. Nursing and midwifery students were compensated with K2000 (US$1). Key informants and clinical instructors were compensated with K5000 (US$3) each. Written informed consent was obtained from each participant prior to the start of the study.

## Results

This study aimed to describe the factors that influence SBE implementation in nursing and midwifery education in Malawi. Quantitative findings demonstrated that 83% of nursing and midwifery students (*n* = 124) and clinical instructors (*n* = 119) had theoretical knowledge of SBE but lacked expertise in its implementation. Five themes emerged from the qualitative data: (1) the dearth of simulation as a teaching strategy in Nurses and Midwives Council of Malawi (NMCM) syllabi for nursing and midwifery programmes; (2) lack of formal training in SBE; (3) SBE demands clinical instructors who are ‘smart’ in knowledge and skills; (4) lack of appropriate human and material resources and (5) old habits die hard.

### Demographic data

Table 2 shows that the study had 149 nursing and midwifery students and 144 clinical instructors who completed the questionnaire. Overall, 215 (73.6%) were female and 78 (26.6%) were male, with 100 (34%) aged 25 years–29 years. On the highest qualification, 123 (41.98%) participants (clinical instructors and final-year students, including master’s students) had a bachelor’s degree majoring in nursing, midwifery, or both nursing and midwifery.

**TABLE 2:** Demographic data for questionnaire respondents (*N* = 293).

Characteristic	Students and clinical instructors		
	Category	*n*	%
Gender	Female	215	73.6
	Male	78	26.6
Age of respondents (years)	15–19	2	0.7
	20–24	74	25.3
	25–29	100	34.1
	30–35	51	17.4
	Over 35	66	22.5
Level of education	Certificates in Nursing, Teaching, Clinical Medicine and Accounting	32	10.92
	MSCE (Ordinary level)	102	34.81
	University Diploma	15	5.12
	Bachelor’s degree	123	41.98
	Master’s degree	21	7.17
Students prior learning	Adult Health Nursing Child Health Nursing Community Health Nursing Mental Health Nursing		

MSCE, Malawi School Certificate of Education.

Focus group discussion participants, who were students, were between 20 years and 35 years, held the Malawi School Certificate of Education, were in the final year of a nursing and midwifery programme, and were all Malawians.

Nursing and midwifery faculty members, clinical instructors, and nursing and midwifery officials from the regulatory body participated in in-depth interviews. They were aged 30 years and above and had more than 6 months of work experience. These participants held bachelor’s (*n* = 17), master’s (*n* = 17) and PhD (*n* = 3) degrees, and all were Malawians. Refer to Table 3 for detailed demographics of the in-depth interview of participants.

**TABLE 3:** Demographics for in-depth interview participants (*N* = 37).

Characteristics	Category	*n*	Relative frequency %
Gender	Male	7	18.9
	Female	30	81.1
Age (years)	30–35	15	40.5
	Over 35	22	59.5
Qualification	Bachelor’s degree	17	45.9
	Master’s degree	17	45.9
	PhD	3	8.2
Profession	Nursing and midwifery faculty members	24	64.8
	Clinical instructors	11	29.7
	Nursing and midwifery officials from the regulatory body	2	5.5
Work experience	6 months – 2 years	8	21.7
	Over 2–5 years	17	45.9
	Over 5 years	12	32.4

### Questionnaire findings

Findings from the questionnaire demonstrated that the majority (83%, *n* = 243) of nursing and midwifery students and clinical instructors had theoretical knowledge of SBE in nursing and midwifery education but lacked skills in its implementation. A total of 13% (*n* = 38) of nursing and midwifery students and clinical instructors mentioned it as their current teaching method. Nursing and midwifery faculty members and clinical instructors highlighted a lack of infrastructure, skills laboratories, teaching hospitals, equipment, inequality in available resources and a lack of formal training as key factors influencing SBE implementation. This necessitated the collection of qualitative data to determine what could be done to equip nursing and midwifery students and clinical instructors with SBE skills. It also necessitated that the study engages clinical instructors on their challenges with the implementation of SBE. It also necessitated a desk review on whether SBE was factored into or included in the regulatory syllabi for nursing and midwifery curricula.

### Qualitative findings

Five themes emerged from the qualitative data, which consisted of individual in-depth interviews and FGDs. Refer to Appendix 3 for the identification of respondents for in-depth Interviews and focus group discussions.

#### Theme 1: The dearth of simulation as a teaching strategy in Nurses and Midwives Council of Malawi syllabi

A desk review conducted at the NMCM Secretariat and in nursing and midwifery training institutions revealed significant gaps in the use of SBE in nursing and midwifery education. These gaps were noticed despite NMCM as the regulatory body of nursing and midwifery education in Malawi having SBE laboratory guidelines. Eight syllabi documents from 17 nursing and midwifery syllabi available at the NMCM were reviewed. These were Bachelors in Child Health Nursing, Adult Health Nursing, Nursing and Midwifery, Nursing and Midwifery Technician (College Diploma), University Certificate in Midwifery, Diploma in Nursing and Midwifery (E-learning), Master of Science in Reproductive Health, and Master of Science in Mental Health. None of the syllabi stipulated SBE as a teaching strategy; rather, syllabi stipulated ‘transformative, innovative, or discovery teaching methods’.

Lecturers from training institutions reported that the NMCM has SBE laboratory guidelines that were developed in 2016, with funding from the International Centre for AIDS Care and Treatment Programs (ICAP) under the Nursing Education Partnership Initiative Project. However, nursing and midwifery training institutions and health facilities neither accessed nor implemented these guidelines. Key informants and a curriculum specialist at the NMCM concurred with participant’s sentiment:

[*I*] don’t think you will find simulation as a teaching method in the syllabi being reviewed … developers of syllabi from various training institutions preferred to stipulate their own transformative, innovative, and discovery teaching methods in the curricula documents. But in 2016, the Council got funding to develop guidelines on simulation, which we have failed to implement in the training institutions to date due to financial constraints … I hope this Simulation Based Education [*SBE*]project will help us review and implement the guidelines in the training institutions.’ (Key Informant 1)

‘If you don’t find simulation in the syllabi, it is because the reviewers of the documents replaced all teaching methods, including simulation, with transformative, innovative and discovery methods, but I have remembered that we have simulation guidelines being sold here, so that you can have a copy.’ (Key Informant 3)

‘There is documentation of simulation as a teaching methodology in our nursing and midwifery curricula. However, there is no detail in the syllabi on how simulation should be utilised during theoretical and clinical teaching sessions. From our experience, we have only used scenarios during OSCE to evaluate student clinical learning but not as a teaching strategy.’ (Key Informant 2)

#### Theme 2: A lack of formal training in simulation-based education

Participants expressed that they had knowledge of SBE but lacked expertise because of a lack of formal training. They acknowledged the existence of knowledge and skill gaps in SBE. Clinical instructors concurred with the sentiments and felt that such gaps could be significantly filled by SBE training of educators and clinical instructors:

‘The majority of us spent our entire college life without being exposed to simulation approach, affecting our skill acquisition. Currently, we have many Continuous Professional Development [*CPD*] sessions at this hospital for members of [*the*] staff but none has been conducted on SBE because the facilitators do not have knowledge and skills on SBE.’ (Key informant 4)

Participants suggested that formal training of nursing and midwifery educators and clinical instructors should be carried out. Establishing SBE centres in training institutions and classrooms in health facilities could promote SBE implementation in Malawi, as indicated by the following two key informants:

‘Simulation training is urgently required to improve skill acquisition. It’s a matter of having simulation centres in training institutions or simulation rooms in health facilities so that students can practice during clinical placement.’ (Key Informant 7)

‘Although the institutions were built some time back there is still room for improvement to accommodate simulation because we need space for students to learn even when they are in the clinical placements. There is a need for collaboration between clinical and training institutions to establish teaching corners. Clinical institutions should find space while training institutions should provide teaching equipment and resources.’ (Key Informant 12)

While some clinical instructors reported that they had never heard of SBE, the majority demonstrated knowledge of SBE, although they lamented their lack of skills. However, the majority felt that SBE could promote skill acquisition among nursing and midwifery students:

‘I have never heard about simulation as a student till now as a nursing administrator. When I see gaps in our students in skill acquisition, I always feel this can be sorted out with the use of simulation.’ (Key Informant 21)

Participants pointed out knowledge and skill gaps in SBE by lecturers and clinical instructors, which they felt significantly influenced their use of SBE in Malawi. They indicated that high workload coupled with a lack of formal training in SBE among educators and clinical instructors also influenced the use of SBE in the training of nurses and midwives in Malawi. This is exemplified in the following quotes:

‘Nurses and midwives have some knowledge in simulation but lack skills on how to use it. The settings do not allow knowledge transfer into skills; this is the major crack in our education system. Educators do not have enough time and exposure to the use of simulation that promotes the transfer of knowledge into practice. It has to start from student preparation for the profession … even if one learns in class, in the clinical area, equipment is improvised, clinical instructors experience a high workload and are not skilled in simulation; how can knowledge be easily transferred and utilised … simulation must be reinforced.’ (Key Informant 25)

‘From experience, most of the clinical preceptors have knowledge and skills for the different nursing and midwifery programmes. However, orienting them to simulation knowledge and skills is key to the transfer of knowledge and skills to students in clinical sites.’ (Key Informant 14)

Participants conclusively indicated that if educator knowledge of simulation was explicitly transferred to curricula documents and reinforced in the acquisition of nursing and midwifery skills during clinical teaching, the improved skills would bring tremendous improvement to the quality of patient care.

**FIGURE 1:**
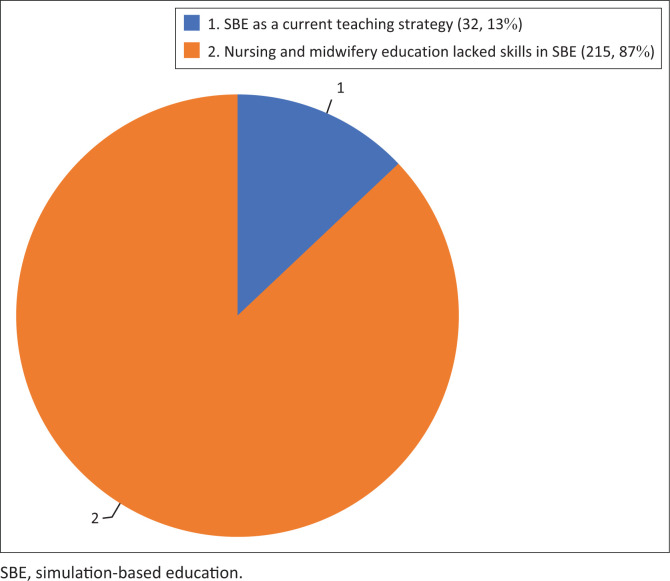
Use of simulation in nursing and midwifery education by nursing and midwifery students and clinical instructors.

The qualitative information presented earlier is also reflected in the collected quantitative survey data. For example, Figure 1 shows that 247 (83.4%) participants reported that they had some knowledge of SBE in nursing and midwifery. However, only 32 (13%) mentioned SBE as a current teaching strategy in training and clinical institutions, and most lacked SBE skills. They reported a lack of hands-on experience in using SBE in clinical teaching.

**FIGURE 2:**
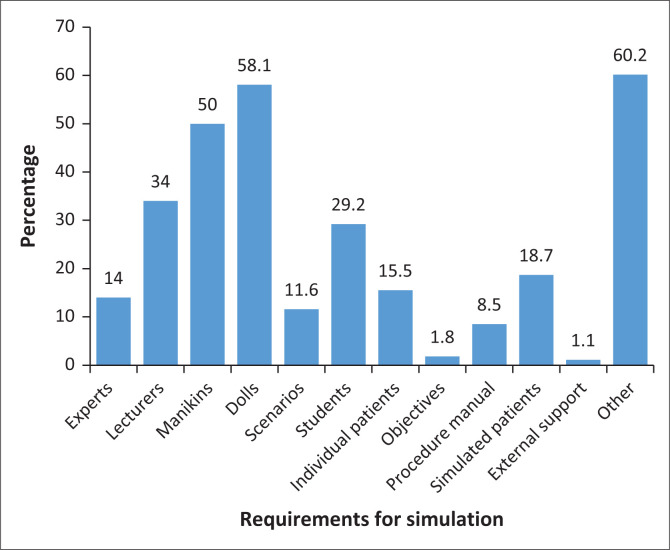
Requirements for simulation.

Similarly, Figure 2 shows that only 5 (2%) mentioned objectives and 33 (12%) mentioned scenarios as requirements for SBE. Over 60% of the participants suggested computers, tablets, virtual reality headsets, other specialised equipment, multimedia content (videos, images and audio), facilities and physical space, seating arrangements conducive to learning, quizzes, exams or other evaluation methods aligned with the simulation learning objectives.

#### Theme 3: Simulation-based education demands clinical instructors who are ‘smart’ in knowledge and skills

The majority of students felt that for effective implementation of SBE in nursing and midwifery education, there is a need for knowledgeable, well-trained and skilled clinical instructors and lecturers in both theory and practice to effectively integrate theory with practice:

‘Nursing and midwifery are both theory and practice [*based*]. We need lecturers and clinical instructors who are “smart” in theory and “smart” in clinical teaching. Some are “half smart.” They teach well in classrooms but are unable to teach us skills in clinical settings. We hope this simulation project will help train our lecturers and clinical instructors to be fully “smart” in theory and practice …’ (Student 3)

‘Nursing and midwifery without skills is not a profession. Teach simulation to faculty members in all training institutions. Maybe we will experience a change in the clinical settings. Some lecturers come for a walk in the wards as they just greet us, register their presence, check objectives for the day, check if their students have found cases, and off they go … thinking they are smart but not in practice. We need not clinical instructors who just say, “What have you done so far?” … yet cannot assist you to perform procedures …’ (Student 9)

‘There is a need for collaborative CPD sessions between facilitators in the training institutions and clinical placement for both cadres to have updated knowledge and skills for practice …’ (Key Informant 30)

#### Theme 4: A lack of resources vis-a-vis large student population is a ‘nightmare’ for simulation-based education

The participants were asked to report the availability of human and material resources by the institution. Most of the resources mentioned were found in training institutions, rather than in clinical settings (Table 4). The most reported available human resources for SBE in the institutions included 75 (56.8%) clinical instructors and 62 (56.8%) nursing and midwifery students. Other notable resources, accounting for 70 (59.8%) of the clinical instructors and respondents nursing and midwifery students combined, encompassed models, CPAP and CPR machines, catheters, cannulas, gloves, nebulisers, internet connectivity and provisions within the skills laboratory. However, few clinical instructors and lecturers reported having scenarios at clinical sites and training institutions, respectively, as shown in Table 4.

**TABLE 4:** Available human and material resources in training and clinical institutions as reported by clinical instructors and nursing and midwifery students (*N* = 293).

Available human and material resources	*n*	Training institution *Frequency*		Clinical institution *Frequency*	
		*n*	%	*n*	%
Internal specialists	109	62	56.8	47	43.1
Lecturers	132	75	56.8	57	43.2
Manikins	7	6	85.7	1	14.3
Dolls	41	30	73.1	11	26.8
Simulation scenarios	11	5	45.5	6	54.5
Student participants	3	3	100	0	0.0
Individual patients	20	14	70	6	30
Simulation objectives	2	1	50	1	50
Others (CPAP, CPR, catheters, cannulas, etc.)	117	70	59.8	47	40.2

CPAP, continuous positive airway pressure; CPR, cardiopulmonary resuscitation.

Participants described the lack of appropriate infrastructure, equipment and teaching facilities with a large student population as a ‘nightmare’ in most training institutions. The participants felt that establishing SBE centres in training institutions and simulation corners in health facilities could alleviate the situation:

‘With inadequate human resources not even skilled in simulation, lack of equipment and teaching health facilities, managing the large student population in congested health facilities is a “nightmare” for most training institutions, and students do not have enough support and time to gain knowledge and practice the skills; hence fail to achieve specific clinical objectives.’ (Key Informant 28)

Some participants felt that, with limited resources in most health facilities and training institutions, clinical instructors and students ended up improvising. They highlighted that this could have a negative impact on the students’ acquisition of skills:

‘During a practical skill on the dressing of an amputated leg, due to lack of appropriate manikin, the lecturer improvises. During check off, it was noted that whatever they had improvised was not ideal and making the students incompetent – meaning that initial setting was not appropriate …’ (Key Informant 13)

‘At this hospital, in terms of resources, we have been furnished with midwifery-related equipment and resources. So, what we need is also equipment for general nursing for the teaching of the other students.’ (Key Informant 17)

The participants’ accounts confirmed that educators and clinical instructors lacked the appropriate resources. Few resources are available in academic training institutions rather than in health facilities. As such, participants reported that they struggled to meet the learning needs of students coming from different training institutions to perform their clinical allocations in health facilities:

‘We need appropriate infrastructure, resources and teaching hospitals for simulation to be ideal in Malawi. Few resources available are in training institutions, not in health facilities.’ (Key Informant 34)

#### Theme 5: Old habits die hard

Most lecturers and students believe that SBE, a significant change in the approach to clinical teaching, poses many challenges for educators and clinical instructors who rely on traditional methods of teaching, such as lecturing. Most proposed that if SBE is to be implemented effectively, it had to be reinforced by NMCM. Otherwise, lecturers, clinical instructors and those considered ‘old-timers’ in nursing and midwifery education systems may be reluctant to adopt SBE:

‘Negativity towards the use of simulation because old habits die hard; some educators or clinical instructors, particularly the old timers in the nursing and midwifery education might not appreciate new ways of doing things; so simulation being a slightly new strategy where lecturers have to come up with scenarios for skill acquisition, use skills laboratories regularly, some lecturers and instructors have some kind of negativity towards it, but NMCM has to reinforce it, and we just have to do it for the sake of students.’ (Key Informant 22)

[*L*]ecturers have their own ways of teaching us in the clinical sites, so bringing change through simulation; they may be reluctant to change, particularly the old timers in the system and those not conversant with some skills unless NMCM reinforces it, they will choose not to do it …’ (Student 7)

[*T*]o bring about change … it is a process. Learning and unlearning take a long time. However, the Continuous Professional Development sessions in simulation will help many lecturers unlearn old ways of teaching and adopt modern teaching methods.’ (Key Informant 19)

This study investigated the determinants shaping the integration of SBE among nursing and midwifery educators and clinical instructors in selected training institutions and clinical sites in Malawi. Survey results revealed that a significant proportion of nursing and midwifery students, along with clinical instructors, possessed theoretical knowledge of SBE as part of their own education, but faced challenges in acquiring practical skills. The qualitative analysis yielded five distinct themes. Nursing and midwifery faculty members and clinical instructors emphasised several crucial factors influencing implementation, including deficiencies in infrastructure, skills laboratories, teaching hospitals, equipment, resource disparities and the absence of formal training opportunities.

## Discussion

This study aimed to identify factors influencing the implementation of SBE among nursing and midwifery lecturers, nursing and midwifery students, and clinical instructors in selected training institutions and clinical sites in Malawi. The discussion relates to the integration of SBE into NMCM syllabi and institutional curricula, and the need for appropriate resources and formal training for effective implementation of SBE.

### Demographics

In the demographic composition of the study participants, a predominant representation was observed among female respondents, predominantly comprising nursing and midwifery students, and clinical instructors. The age distribution indicated a concentration within the 25–29 age bracket. Furthermore, a significant proportion of participants held bachelor’s degrees, suggesting a high educational attainment level within the sampled cohort. These demographic features provide a nuanced understanding of the profile of the respondents, offering insights into the gender distribution, age range and educational qualifications of the study population.

### The dearth of simulation as a teaching strategy in Nurses and Midwives Council of Malawi syllabi

The study’s findings showed significant knowledge of SBE by nursing and midwifery students and clinical instructors, and that SBE was not a new clinical teaching strategy in the Malawian context. Li et al. (2022) found that using SBE in nursing and midwifery education was not new in low resource settings. Although most participants in this study reported that they knew about SBE, findings demonstrated that none of the nursing and midwifery syllabi developed for the regulatory body of nursing and midwifery education in Malawi stipulated SBE as a teaching strategy, apart from merely stipulating transformative, innovative and discovery teaching methods. Currently, there are no universal SBE guidelines that could be used by educators and students as clinical standards in clinical skills laboratories, training institutions and clinical settings or to guide the implementation of SBE in health facilities in Malawi. The results confirm findings from prior studies which show that even though many global nursing educational accrediting bodies use SBE for licensure examinations, no standard guidelines have been proposed for the implementation of SBE (Janse et al. 2018; Puri et al. 2017).

### A lack of formal training in simulation-based education

Formal training programmes in SBE have become a pivotal component in preparing educators and healthcare professionals for their roles (Greenwood & Ewell 2018). These programmes are designed to equip individuals with the necessary skills and knowledge to effectively utilise simulation techniques in both educational and clinical settings (Pitriani et al. 2019). Participants in this study felt that SBE training could significantly help in designing realistic and meaningful simulation scenarios that mirror real-world situations. Several studies have shown that the existence of formal training programmes in SBE reflects a growing recognition of the transformative potential of implementing SBE as a clinical teaching strategy (Lainie & Oriol 2022; Greenwood & Ewell 2018; Koukourikos et al. 2021). This skill is crucial for ensuring that learners, whether students or healthcare providers, are exposed to relevant and challenging experiences that promote skill acquisition and critical thinking.

### A lack of appropriate human and material resources vis-à-vis large student population is a ‘nightmare’ for simulation-based education

A lack of appropriate material resources and large student population in relation to clinical settings were also reported as critical issues influencing SBE implementation in all training and clinical institutions in Malawi. The decreased quality of clinical teaching and patient care has been attributed to knowledge gaps, staff shortages with high workloads, skill decline, loss of motivation, lack of appropriate infrastructure and equipment, and demanding inclusion of SBE in health-related curricula (Angelina, Stephen & Ipyana 2021; Fritz et al. 2020; Nelissen et al. 2017). In this study, despite being obtained locally, the most basic infrastructure and equipment resources available in training institutions were reported as inappropriate, inadequate or technologically outdated. A lack of material resources prevents students from adequately using SBE and evidence-based methods, which they may have learned in class during theory (Song & Jang 2021).

### Simulation-based education demands clinical instructors who are ‘smart’ in knowledge and skills

Most clinical instructors in this study reported a lack of skill and expertise in SBE, which significantly affected the use of this strategy in working with students and staff. Despite having theoretical knowledge of SBE, it was evident that they could not translate the knowledge into nursing and midwifery practice because few educators or students had named SBE a current teaching strategy. Botma (2014) emphasised that nursing and midwifery students should be able to transfer theoretical knowledge during clinical practicum while in training because health services demand that those exiting a training programme be competent at the entry level. The inability of nurses and midwives to transfer what they have learned in class to the clinical setting has been deemed the failure of educators to use teaching strategies that promote the transfer of learning. This necessitates innovative teaching and learning strategies, such as SBE in nursing and midwifery pedagogy, through formal training.

Furthermore, the findings of this study demonstrate that most SBE equipment is expensive and is a barrier to its use. Evidence from high-income countries indicates that it may not be feasible to implement SBE using high-technology simulators in low-income countries because of prohibitive costs (Puri et al. 2017). Simulation-based education in low-income countries can be effectively implemented using low-fidelity simulators, simulated patients and scenarios, and adequate training to enhance knowledge and skill development (Jacobs & Van Jaarsveldt 2016; Janse et al. 2018, 2021). In addition, the involvement of simulated patients in scenario development enhances the authenticity of SBE, because an understanding of the nuances of simulation scenarios helps educators, students and clinical instructors to create an immersive and safe learning environment (Abulebda, Auerbach & Limaiem 2023; Bienstock & Heuer 2022; Chernikova et al. 2020). As the demand for skilled educators and competent healthcare providers continues to rise, the role of formal training in SBE is expected to expand further.

### Old habits die hard

The term ‘old habits’ implies deeply ingrained practices that may be resistant to change, underscoring the challenge faced in introducing a more contemporary and experiential teaching method. The identified theme, ‘Old Habits Die Hard’, encapsulated a crucial aspect of the study’s findings, pointing to a prevailing resistance among faculty members towards embracing SBE as a transformative approach to training nursing and midwifery students. This resistance highlights the reluctance to depart from conventional teaching methodologies and adapt to innovative pedagogical practices (Cheraghi et al. 2023). The theme underscores the entrenched nature of traditional teaching habits within the nursing and midwifery education landscape, suggesting hesitancy among faculty members to adopt SBE as a vehicle for pedagogical transformation.

Participants recognised the potential of SBE to transform clinical education. However, the resistance noticed among faculty members indicates a substantial barrier to the use of SBE and its transformative potential. Such resistance could be rooted in various factors such as a lack of familiarity with SBE methodologies, apprehensions about their effectiveness, or a preference for traditional teaching paradigms (Baayd et al. 2023). These findings suggest the need for targeted interventions to address this resistance, including professional development opportunities, awareness campaigns and initiatives aimed at highlighting the tangible benefits and outcomes of SBE. Addressing these ingrained habits and fostering a mindset shift among faculty members are crucial for the successful integration and optimisation of SBE within nursing and midwifery training programmes.

### Strengths and limitations

Utilising a mixed-methods design to investigate the factors shaping SBE within nursing and midwifery training programmes in Malawi provided a comprehensive and flexible research approach, harnessing the distinct advantages of quantitative and qualitative research methodologies. This integrative methodology not only enabled the quantification of measurable variables but also delved into the nuanced perspectives, experiences and perceptions of the participants, thereby offering a holistic understanding of the multifaceted dynamics inherent in SBE. By seamlessly blending numerical data with rich narrative insights, this approach contributed to the robustness of the study, fostering a more nuanced and insightful exploration of the complex interplay between contextual and educational factors that influence the effectiveness and implementation of simulation-based strategies in the specific context of nursing and midwifery training in Malawi. However, the limitations of this study include the fact that it was conducted in training institutions and health facilities in which researchers have constant interaction with the educators, students and clinical instructors who participated in the study. To some extent, this might have made external validation difficult. Data were collected from one district hospital, four referral hospitals and five training institutions. This may have affected the quality of the data collected and the generalisability of the findings.

### Recommendations

The findings from this study underscore the need to develop targeted professional development initiatives for educators to enhance SBE integration. Furthermore, SBE resources need to be prioritised in budgets to include the training of faculty and clinical preceptors, as well as equipment acquisition. Finally, there is a need to incorporate SBE into nursing and midwifery curricula in both teaching strategies and student engagement.

## Conclusion

Appropriate infrastructure, adequate equipment, and knowledgeable and skilled nursing and midwifery educators and clinical instructors are essential for the effective implementation of SBE in Malawi. Educators need training and experience to design and deliver SBE scenarios that accurately reflect real-world situations. Increased access to appropriate infrastructure in both training institutions and health facilities is needed to fully realise the benefits of SBE as a clinical teaching strategy.

Providing high-quality professional training to prepare nursing and midwifery students for real-world scenarios can be challenging without access to these resources. In conclusion, improving the realistic perception of SBE in Malawi requires a multifaceted approach that addresses the need for appropriate infrastructure, sufficient resources, knowledgeable and skilled educators, and clinical instructors. By addressing these factors, nursing and midwifery students in Malawi can receive high-quality training to prepare them for challenges in professional practice.

## Appendix 1: Interview guide for key informants use of simulation-based education

### Interview Guide

*For individual interviews.*

Institution Region of the country Interview

Code

Researcher Initials

Day/Month/Year of interview:

### SECTION II: KNOWLEDGE, EXPERIENCES, CHALLENGES AND OPERATIONAL STRATEGIES ON SIMULATION-BASED EDUCATION IN NURSING AND MIDWIFERY.

*Now I would like to know more about your knowledge, experiences, challenges (if any) about use of simulation in Nursing and Midwifery Education*

**1. Can you share with me the current teaching methodology in nursing and midwifery education in Malawi?**

*Probe on:*

- *Perceptions/thoughts on the current teaching methodology upon student learning.*

**2. Can you share with me your understanding of simulation as a pedagogic method in nursing and midwifery education?**

*Probe on:*

- *Sources of knowledge on use of simulation-based education as a pedagogic method.*
- *Difference between simulation and other teaching methods.*
- *Requirements to effectively conduct a nursing or midwifery class on simulation.*
- *How can simulation be used?*

**3. What do you think could be the benefits of using simulation-based education in nursing and midwifery?**

**4. Can you share with me your experience (if any) of using simulation in nursing and midwifery education as a student/a clinical instructor/an educator**?

*Probe on:*

- *Sources of experience on simulation.*
- *Type/nature of experience in the skills lab and in the clinical setting.*
- *Advantages of using simulation in the training of nurses and midwives.*
- *How can use of simulation stimulate critical thinking and problem-solving skills?*

**5. What do you think are the gaps in the curriculum that the use of simulation would fill in nursing and midwifery education in Malawian setup?**

*Probe on:*

- *Gaps in clinical t eaching*
- *Gaps in nursing skills and communication skills*
- *Gaps in clinical settings*
- *Others*

**6. What could be some of the challenges in using simulation in nursing and midwifery education in Malawi**?

*Probe on:*

- *Challenges in nursing and midwifery education*
- *How equipped are the nursing and midwifery training institutions for simulation-based education?*
- *Expertise of nursing and midwifery educators to teach simulation*
- *Effect/impact of large classes on the use of simulation*
- *Challenges in the clinical settings*
- *Others*

**7. What do you think needs to be done to introduce or improve the use of simulation in nursing and midwifery education in Malawi**?

*Probe on:*

- *Curriculum development*
- *In training institutions*
- *In clinical settings*
- *Roles of institutional management and nurse administrators*
- *Roles of Nursing and Midwifery Directorate, MOH*
- *Roles of Nurses and Midwives Council of Malawi*

**8. Do you have any final comments or suggestions on how simulation in nursing and midwifery training institutions can be improved?**

**Concluding remarks**

Thank you so much for sharing your opinions with me. If you have any further queries do feel free to get in touch with me through the number I have provided.

## Appendix 2: Guide for focus group discussions with final-year students’ use of simulation-based education the body map

Respondent groups: Nursing and Midwifery students in final year of the programme

Purpose: To explore gaps and feasibility of simulation-based education in nursing and midwifery programmes in Malawian context.

### I. Introduction

*We are interested in exploring use of simulation-based education in nursing and midwifery education.*

### II. Drawing a healthy/happy student

1. On flip-chart, ask the participants to draw a picture of a typical student in college within three teaching settings (classroom, skills lab, clinical site) – what makes her/him happy?)
2. When the drawing is finished, give the participants three small pieces of paper and ask them to write two common teaching methods taking place in each teaching setting**/indicating their preferred teaching method for each setting** (one teaching setting per piece of paper).
3. When they have finished, ask each participant to read aloud the teaching methods, his/her preferred teaching method per setting and place them on relevant teaching setting on the drawing/picture. It does not matter if some teaching methods are repeated.

### III. Use the questions below to facilitate a discussion:

1. Can you share with me the common teaching methods in nursing and midwifery education at this institution?
  *Probe on:*
  - *Common teaching methods in classroom, skills lab and clinical settings.*
  - *Perceptions/thoughts on the current teaching methods upon student learning.*
2. Can you share with me your understanding of simulation as a pedagogic method in nursing and midwifery education?
  *Probe on:*
  - *Sources of knowledge on use of simulation-based education as a pedagogic method.*
  - *Difference between simulation and the other mentioned teaching methods.*
  - *Requirements to effectively conduct a nursing or midwifery class on simulation.*
  - *How can simulation be used?*
3. What do you think could be the benefits of using simulation-based education in nursing and midwifery over the other mentioned teaching methods?
4. Can you share with me your experience (if any) of using simulation in nursing and midwifery education as a student?
  *Probe on:*
  - *Sources of experience on simulation.*
  - *Type/nature of experience in the skills lab and in the clinical setting.*
  - *Advantages of using simulation in nursing and midwifery practice.*
  - *How can use of simulation stimulate critical thinking and problem-solving skills?*
5. What do you think are the gaps in the curriculum that the use of simulation would fill in nursing and midwifery education at this institution?
  *Probe on:*
  - *Gaps in clinical teaching*
  - *Gaps in nursing skills and communication skills*
  - *Gaps in clinical settings*
  - *Others*
6. What could be some of the challenges in using simulation in nursing and midwifery education at this institution?
  *Probe on:*
  - *Challenges in classroom, skills lab and clinical setting.*
  - *How equipped is the nursing and midwifery training institution for simulation-based education?*
  - *Expertise of nursing and midwifery educators to teach simulation.*
  - *Effect/impact of large classes on the use of simulation.*
  - *Others*
7. What do you think needs to be done to introduce or improve the use of simulation in nursing and midwifery education in Malawi?
  *Probe on:*
  - *Curriculum development*
  - *At the training institution*
  - *In clinical settings*
  - *Roles of institutional management and nurse administrators*
  - *Roles of Nursing and Midwifery Directorate, MOH*
  - *Roles of Nurses and Midwives Council of Malawi*
8. Do you have any final comments or suggestions on how simulation in nursing and midwifery training institutions can be improved?

**Concluding remarks**

Thank you so much for sharing your opinions with me. If you have any further queries do feel free to get in touch with me through the number I have provided.

## Appendix 3

**TABLE 1-A3:** Identification of respondents for In-depth Interviews and focus group discussions.

Participant	Identification	Roles
Key Informant #1	Education Specialist, NMCM	Manages Nursing and Midwifery curricula
Key Informant #3	Registrar, NMCM	Manages NMCM
Key Informant #2	Dean of School, KUHeS, Blantyre Campus	Manages an academic school
Key Informant #4	Clinical Instructor, Nkhotakota District Hospital	Teaches students in the clinical area
Key Informant #7	Clinical Instructor, Queen Elizabeth Central Hospital	Teaches students in the clinical area
Key Informant #12	Clinical Instructor, Kamuzu Central Hospital	Teaches students in the clinical area
Key Informant #21	Clinical Instructor, Zomba Mental Hospital	Teaches students in the clinical area
Key Informant #25	Clinical Instructor, Mzuzu Central Hospital	Teaches students in the clinical area
Key Informant #14	Clinical Instructor, Mzuzu Central Hospital	Teaches students in the clinical area
Student #3	Student, KUHeS Lilongwe Campus	Trainee at an academic institution
Student #9	Student, Malawi College of Health Sciences, Blantyre	Trainee at an academic institution
Key Informant #30	Clinical Instructor, Kamuzu Central Hospital	Teaches students in the clinical area
Key Informant #28	Clinical Instructor, KUHeS, Lilongwe Campus	Teaches students in the clinical area
Key Informant #13	Clinical Instructor, St. John of God Nursing College’ Mzuzu	Teaches students in the clinical area
Key Informant #17	Clinical Instructor, Mzuzu Central Hospital’	Teaches students in the clinical area
Key Informant #34	Lecturer, KUHeS Lilongwe Campus	Teaches students in training institutions
Key Informant #22	Lecturer, KUHeS BT	Teaches students in training institutions
Student #7	Student, KUHeS, Lilongwe Campus	Trainee at an academic institution
Key Informant #19	Head of Department, Mzuzu University	Manages an academic department
